# Genetic overlap between type 2 diabetes and depression in Swedish and Danish twin registries

**DOI:** 10.1038/mp.2016.28

**Published:** 2016-03-29

**Authors:** C Kan, N L Pedersen, K Christensen, S R Bornstein, J Licinio, J H MacCabe, K Ismail, F Rijsdijk

**Affiliations:** 1 Institute of Psychiatry, Psychology and Neuroscience, King's College London, London, UK; 2 Department of Medical Epidemiology and Biostatistics, Karolinska Institute, Stockholm, Sweden; 3 The Danish Twin Registry, University of Southern Denmark, Odense, Denmark; 4 Department of Clinical Genetics and Department of Clinical Biochemistry and Pharmacology, Odense University Hospital, Odense, Denmark; 5 Department of Diabetes, King's College London, London, UK; 6 Medical Department and Clinic III, Carl Gustav Carus University Hospital, Dresden, Germany; 7 South Australian Health and Medical Research Institute, Adelaide, SA, Australia; 8 Flinders University, Adelaide, SA, Australia

## Abstract

A bidirectional association between type 2 diabetes (T2DM) and depression has been consistently reported. Depression is associated with worse biomedical outcomes and increased mortality. The mechanisms underlying the association of T2DM with depression remain unclear. One possible question we can address is the extent to which the co-occurrence of diabetes and depression is due to correlated genetic and/or environmental risk factors. In this study, we performed structural equation model fitting to population-level data from the Swedish (*n=*68 606) and Danish (*n=*95 403) twin registries. The primary outcomes were clinical diagnosis of T2DM and depression using national hospital discharge registries. The phenotypic correlation between T2DM and depression is modest in both samples. In the Swedish sample, unique environmental effects explain a greater proportion of the covariance in males, whereas the association is primarily attributed to genetic effects in females. In the Danish sample, genetic effects account for the majority of the covariance in both males and females. Qualitative genetic sex differences are observed in both samples. We believe this is the first study to demonstrate significant genetic overlap between T2DM and depression.

## Introduction

Cross-sectional and longitudinal studies have consistently reported an association between type 2 diabetes (T2DM) and depression,^1, 2^ with up to 60% increased risk for developing T2DM in individuals with depression and 15% increased risk for incident depression in those with T2DM.^3, 4^ Depression has been associated with adverse effects on diabetes outcomes including suboptimal glycaemic control, complications and higher rates of mortality. Tentative evidence suggests a common biological pathway between T2DM and depression, as people with depression have increased levels of inflammation, hyperactivity in the hypothalamic–pituitary adrenal axis and sympathetic nervous system.^5^ Those biological processes also underlie T2DM.^6^

A significant genetic correlation between T2DM and depression would provide evidence in support of a common genetic pathway to both disorders. Although it is known that genetic factors are aetiologically important in both T2DM and depression, it is unclear to what extent the T2DM-depression association correlated genetic or environmental factors. Twin studies provide a valuable approach to investigate genetic influences on complex traits or disorders,^7^ as well as the genetic architecture of comorbidity. They use the similarity within and between diseases across monozygotic (MZ) and dizygotic (DZ) twins to estimate the relative importance of the effects of genetic, shared and individual-specific environmental factors on the variance and covariance of the disorders.

Two twin studies have addressed the genetic comorbidity of T2DM and depression. The Vietnam Era Twin Study of Aging reported a significant odd ratio between T2DM and depression (1.7 (95% confidence interval: 1.1–2.7)) in a sample restricted to males aged 50–59. They concluded that there was no significant genetic correlation (0.19 (0–0.46)) or unique environmental correlation (0.09 (0–0.45)) between T2DM and depression.^8^ A study based on the Screening Across the Lifespan Twin (SALT) study reported a significant phenotypic correlation (0.20 (0.09–0.33)) in combined analyses of male and female twins aged 40 years and older. They concluded that there was significant environmental correlation (0.54 (0.02–0.88)) between T2DM and depression.^9^

Although current evidence does not support a common genetic pathway hypothesis, a genetic model that incorporates possible quantitative and qualitative sex differences in genetic and environmental effects is called for, given the substantially higher prevalence rate of comorbid T2DM and depression in females compared with males.^10, 11, 12^ We therefore aimed to examine the genetic aetiology of the T2DM-depression association in two large population samples of twins using sex-limitation genetic modelling. In this study we combined the data from the Swedish and Danish twin registries, as they have a similar ethnic population structure.

## Materials and methods

### Sample

The Swedish Twin Registry (http://ki.se/twinreg) covers over 99% of all twins born in Sweden between 1886 and 2000 (~160 000 individuals).^13^ This study will include data from the following two cohorts: (i) SALT (*n=*44 113) and (ii) Swedish Twin Studies of Adults: Genes and Environment (STAGE; *n=*24 493). The SALT cohort consists of twins born between 1886 and 1958,^13, 14^ whereas the STAGE cohort consists of twins born between 1959 and 1985.^15^ The project was approved by the Regional Research Ethics Board at Karolinska Institute and the Steering Board of the Swedish Twin Registry.

The Danish Twins Registry covers twin births in Denmark since 1870, with 170 000 individuals registered as of January 2011.^16^ This study will include all birth cohorts (1870–1930, 1931–1952, 1952–1982 and 1983–2000), excluding participants aged <18 at time of data retrieval. The project was approved by the Danish Data Protection Agency.

### Primary variables from hospital registry records

The Swedish National Patient Registry has coverage of inpatient care for medical and psychiatric disorders since 1963 on a regional basis, extended to complete coverage in 1987 and further expanded to include outpatient care in 2001. Disorders were coded according to the International Classification of Disease (ICD) 8th, 9th or 10th Editions. The Swedish Twin Registry is linked to the National Patient Registry using a unique personal registration number assigned to all residents of Sweden. Data were extracted from 1969 to 31 December 2010.

The Danish National Patient Register initially had coverage of inpatient care for somatic disorders in 1977, and extended to complete coverage of all inpatient and outpatient care for medical and psychiatric disorders since 1995.^17^ Disorders were coded according to the ICD 8th or 10th Editions. The Danish Twin Registry is linked to the National Patient Registry using a unique personal identification number recorded in the Danish Civil Registration System. Data were extracted from 1977 to 1 April 2011.

The primary outcomes are ICD diagnoses of T2DM and depression from the respective National Hospital Discharge Registry, defined a prior to maximise statistical power. T2DM is defined by the following codes: (i) ICD-8: 250; (ii) ICD-9: 250.00, 250.02, 250.10, 250.12, 250.20, 250.22, 250.30, 250.32, 250.40, 250.42, 250.50, 250.52, 250.60, 250.62, 250.70, 250.72, 250.80, 250.82, 250.90, 250.92 and (iii) ICD-10: E11. Depression is defined by the following codes: (i) ICD-8: 296.0, 296.2, 296.9, 298.0, 300.4; (ii) ICD-9: 296.2, 296.3, 300.4, 301.10, 301.12, 301.13, 309.10, 311 and (iii) ICD-10: F32, F33, F34.1 and F38.1 (Supplementary Table 1).

### Secondary variables

Zygosity of same-sex twin pairs was based on standard self-report items and, when validated with biological makers, has an accuracy of 95–99% in the Swedish Twin Registry^18^ and 96% in the Danish Twin Registry.^19^ Demographic details included age and sex. The demographic data were retrieved on 31 December 2010 for the Swedish twin sample and 11 October 2014 for the Danish twin sample.

### Statistical analysis

#### Genetic model fitting

The classical twin method is based on comparison of MZ twins who share 100%, whereas DZ twins share on average 50% of segregating genes. In the univariate ACE model, individual differences in a trait are assumed to arise from: (i) additive genetic influences (A), (ii) shared environmental influences (C) and (iii) unique environmental influences (E). In bivariate twin analysis, in addition to the variance components of the individual traits, the main goal is to decompose the phenotypic correlation between them into parts due to correlating addictive genetic (rA), shared environmental (rC) and unique environmental (rE) effects predisposing to both traits.^7^ Quantitative sex differences are modelled by specifying sex-specific ACE paths for T2DM and depression as well as rA, rC and rE.^20^ The power is derived from different MZ-DZ correlation ratios across sex. Qualitative sex differences are incorporated by specifying free correlational paths between (i) all male and female genetic factors or (ii) all male and female common environmental factors in DZ opposite-sex twin pairs. The power is derived from differential within-trait and cross-trait correlations in opposite-sex compared with same-sex DZ pairs.^20^

Genetic model fitting analysis was performed in the programme OpenMx.^21^ A liability threshold model was used, assuming that the risk to T2DM and depression is each normally distributed in the general population with the disorder manifesting when a certain threshold of risk is exceeded.^22^ Their joint distribution is assumed to have a bivariate normal distribution, with the relative proportions of concordant (both twins above or below the thresholds) and discordant pairs informing on the correlation between the liabilities. Given the range of birth year from 1896 to 1985 in the Swedish Twin Registry and from 1870 to 2000 in the Danish Twin Registry, age at the time when the demographic data were retrieved was modelled as a covariate on the thresholds.

Two criteria were used to choose the best-fitting, parsimonious model: (i) differences in minus twice the log-likelihood (−2LL) yielding a statistic equivalent to a *χ*^2^-test, with degrees of freedom equal to the difference in the numbers of parameters and (ii) Akaike's Information Criterion (AIC),^23^ with lower values indicating a better balance between explanatory power and parsimony. A difference in AIC at least 10 indicates substantial support in favour of the more parsimonious model.

#### Secondary analysis

The genetic model fitting analysis was repeated and restricted to individuals born after 1950 in both the Swedish and Danish samples.

## Results

### Descriptive finding

The Swedish Twin Registry from the SALT and STAGE cohorts consists of 68 606 twin individuals with known age, sex, zygosity, T2DM and depression. This results in 50 082 twin pairs and 18 524 single twins (Table 1). The mean age was 58.5 (17.6) as of 31 December 2010 when the demographic data were retrieved.

The Danish Twin Registry consists of 115 886 twin individuals, of whom 95 403 individuals have known age, sex, zygosity, T2DM and depression status and are aged over 18. This results in 94 194 twin pairs and 1209 single twins (Table 1). The mean age was 60.0 (16.8) as of 11 October 2014 when the demographic data were retrieved.

In both the Swedish and Danish samples, the prevalence rates for T2DM and depression were similar for males and females (Table 1). Mean-centred age was incorporated as a covariate, and thus, the thresholds and other estimates apply to a sample at mean age. To illustrate, the prevalence rate for T2DM in males for the Swedish sample is 4.7% (*z*-value=1.67) at age 59. With the effect of age being estimated at –0.03, T2DM prevalence rates will be 1.3% at age 40 (*z*-value=2.24) and 9.0% at age 70 (*z*-value=1.34).

Logistic regression analysis revealed a positive relationship between T2DM and depression in males and females in the Swedish (Rph: males 0.13 (0.08–0.14); females 0.16 (0.12–0.17) and Danish (Rph: males 0.16 (0.12–0.20); females 0.15 (0.12–0.20)) samples. Tetrachoric correlations stratified by zygosity and sex are summarised in Supplementary Table 2.

### Genetic model fitting in the Swedish twin sample

The starting point was an ACE model that included both quantitative and qualitative sex differences in genetic factors (model IS: −2LL=52 061.97; df=137 186; AIC=−222 310). The effect of common environment was negligible, shown by a non-significant decline in fit of the AE model (model 2S: −2LL=52 063.00; df=137 192; AIC=−222 321; *χ*^2^=1.04; *P=*0.98). To test the significance of qualitative sex differences, all genetic factors were constrained to correlate at 0.50 across males and females in opposite-sex pairs model, resulting in a significantly poorer fit of the AE model (model 3S: −2LL=52 116.61; df=137 196; AIC=−222 275; *χ*^2^= 53.61; *P<*0.05). All A and E paths are therefore estimated freely for males and females with no further constraints, as it was not possible to equate them without a significant worsening of fit.

**Figure 1:**
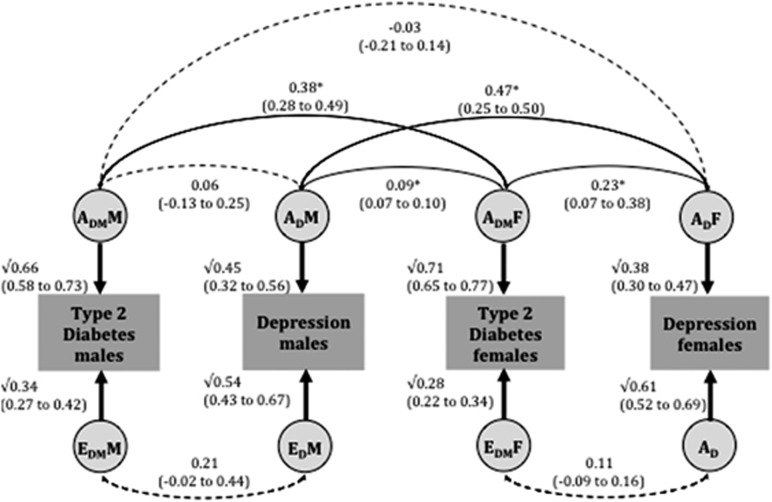
Parameters estimates from bivariate AE twin models for type 2 diabetes and depression, using the Swedish twin sample, with age as a covariate. Best-fit full sex-limitation bivariate AE model fit to Swedish data in opposite-sex dizygotic twins. Asterisk indicates a significant pathway. A indicates addictive genetic effects; E for unique environmental effects; subscript _DM_ for type 2 diabetes; subscript _D_ for depression DM; M for males and F for females. Additive genetic and unique environment contributions to type 2 diabetes and depression are indicated by A_DM_M, A_D_M, E_DM_M and E_D_M in males, and by A_DM_F, A_D_F, E_DM_F and E_D_F in females, respectively.

The best-fitting model is therefore the full sex-limitation bivariate AE model (Figure 1), with both quantitative and qualitative sex differences. The heritability estimates for clinical diagnoses of T2DM and depression were 66% (58–73%) and 45% (32–56%) in males and 71% (65–77%) and 38% (30–47%) in females, respectively. The genetic correlation between T2DM and depression was non-significant in males (0.06 (−0.13–0.25)) but significant in females (0.23 (0.07–0.38)). In males, the phenotypic correlation between T2DM and depression was 31% due to genetic factors and 69% due to individual-specific environmental factors, whereas in females, these values were 75% and 25%, respectively.

Under the assumption of random mating, all correlations between genetic factors are expected to be 0.50 in same-sex DZ sibling pairs.^24^ The estimated correlation between male and female genetic factors for T2DM (A_DM_M and A_DM_F) was 0.38 (0.28–0.49) and for depression (A_D_M and A_D_F) is 0.47 (0.25–0.50; Figure 1). They are close to the expected value of 0.50, suggesting little or no qualitative sex difference within diabetes and depression, respectively. The estimated correlations between the genetic factors for (i) male T2DM and female depression (A_DM_M and A_D_F) and (ii) male depression and female T2DM (A_D_M and A_DM_F) were −0.03 (−0.21–0.14) and 0.09 (0.07–0.10), respectively. They are substantially different from 0.50, indicating that different genetic factors are at play in males and females when it comes to explaining the T2DM-depression association (qualitative sex difference).

### Genetic model fitting in the Danish twin sample

The same approach was adopted in the Danish sample. The starting model was the ACE model with both quantitative and qualitative sex differences in genetic factors (model ID: −2LL=58 776.30; df=176 878; AIC=−294 979.7). The effect of common environment was again negligible, as shown by a non-significantly decline in fit (model 2D: −2LL=58 778.83; df=176 884; AIC=−294 989.2; *χ*^2^=2.52; *P=*0.87). The effect of qualitative sex difference was important, as when all genetic factors were constrained to correlate at 0.50 across males and females in the opposite-sex pair model, a significantly poorer fit of the AE model resulted (model 3D: −2LL=58 838.40; df=176 888; AIC=−294 937.6; *χ*^2^=62.10; *P<*0.05). All A and E paths are therefore estimated freely for males and females with no further constraints.

**Figure 2:**
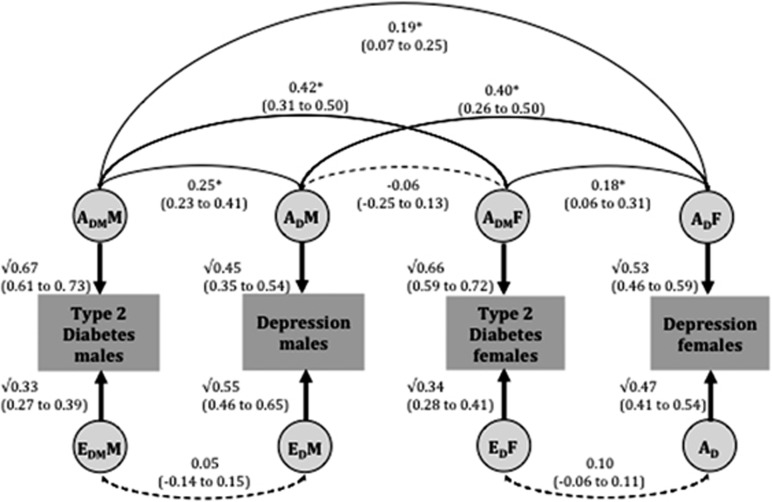
Parameters estimates from bivariate AE twin models for type 2 diabetes and depression, using the Danish twin sample, with age as a covariate. Best-fit full sex-limitation bivariate AE model fit to Danish data in opposite-sex dizygotic twins. Asterisk indicates a significant pathway. A indicates addictive genetic effects; E for unique environmental effects; subscript _DM_ for type 2 diabetes; subscript _D_ for depression DM; M for males and F for females. Additive genetic and unique environment contributions to type 2 diabetes and depression are indicated by A_DM_M, A_D_M, E_DM_M and E_D_M in males, and by A_DM_F, A_D_F, E_DM_F and E_D_F in females, respectively.

The best-fitting model is also the full sex-limitation bivariate AE model (Figure 2), with both quantitative and qualitative sex differences. The heritability estimates for clinical diagnoses of T2DM and depression were 67% (61–73%) and 45% (35–54%) in males and 66% (59–72%) and 53% (46–59%) in females, respectively. In both males and females, the genetic correlation between T2DM and depression was moderate and significant (males: 0.25 (0.23–0.41); females: 0.18 (0.06–0.31)). The phenotypic correlation was primarily attributed to shared genetic effects in both sexes (males: 87% females: 74%).

The estimated correlation between male and female genetic factors for T2DM (A_DM_M and A_DM_F) was 0.42 (0.31–0.50) and for depression (A_D_M and A_D_F) was 0.40 (0.26–0.50; Figure 1). They are close to the expected value of 0.50, suggesting little or no qualitative sex difference within diabetes and depression, respectively. The estimated correlations between the genetic factors for (i) male T2DM and female depression (A_DM_M and A_D_F) and (ii) male depression and female T2DM (A_D_M and A_DM_F) were 0.19 (0.07–0.25) and −0.06 (−0.25–0.13), respectively. These are substantially different from 0.50, indicating that different genetic factors are involved in explaining the T2DM-depression association across sex (qualitative sex difference).

### Secondary analysis

In samples restricted to individuals born after 1950, the best-fitting model is also the full sex-limitation bivariate AE model in both the Swedish and Danish samples, with both quantitative and qualitative sex differences (data not shown). In the Swedish sample, the phenotypic correlation between T2DM and depression was significant in both males (0.21 (0.13–0.30)) and females (0.25 (0.17–0.33)). The heritability estimates for T2DM and depression were 73% (58–81%) and 52% (50–67%) in males and 72% (58–84%) and 40% (30–49%) in females, respectively. The genetic correlation between T2DM and depression was significant for females (males: 0.00 (−0.24–0.32); females: 0.49 (0.26–0.73)). There is no major difference between the findings from the primary and secondary analysis.

In the Danish sample, the phenotypic correlation between T2DM and depression was significant in both males (0.11 (0.02–0.19)) and females (0.13 (0.04–0.22)). The heritability estimates for T2DM and depression were 78% (67–85%) and 45% (31–58%) in males and 78% (62–89%) and 47% (36–57%) in females, respectively. The genetic correlation between T2DM and depression was no longer significant (males: 0.07 (−0.18–0.32); females: 0.21 (−0.06–0.47)), in comparison with the primary analysis, as the 95% confidence intervals cross zero.

## Discussion

To the best of our knowledge, this is the first study to examine the genetic aetiology of the T2DM-depression comorbidity using hospital registry data from two large population samples of male and female twins covering the entire adult age range. The most notable feature of our finding is the similarity between the results from the Swedish and Danish samples. Both samples support the notion that qualitative genetic sex differences are at play in the genetic overlap between T2DM and depression. The main difference is the proportion of the T2DM-depression association due to shared genetic factors between Swedish and Danish samples. In the Swedish sample, the phenotypic relationship is mainly due to unique environment factors in males and shared genetic factors in females. In the Danish sample, it is due to shared genetic effects in both sexes. The observations were attenuated in the analyses restricted to individuals born after 1950. Although this could be a genuine difference, the 95% confidence intervals around the genetic correlations are overlapping between the primary and secondary analyses. In addition, the secondary analysis is half of the initial sample size for the Swedish sample (primary analysis: *n=*68 606; secondary analysis: *n=*35 912) and two fifths for the Danish sample (primary analysis: *n=*95 403; secondary analysis: *n=*40 984), leading to reduced power. The secondary analysis further strengthens the validity of our primary findings, as any observed cohort effect may potentially be secondary to multiple residual confounders. The findings, however, did not differ significantly between the entire sample and those born after 1950, especially in the Swedish sample.

### Previous literature

Our results differ from previous findings. The Vietnam Era Twins Study of Aging^8^ suggests that there is no evidence of common genetic factors in contributing to the T2DM-depression association. The finding is difficult to interpret as it was restricted to males aged 50–59, relied on a much smaller sample and used self-reported data to construct the diagnosis of T2DM and depression.

The SALT study (using a cohort subsample included in our Swedish sample) previously reported a significant contribution of unique environmental factors in the T2DM-depression association, but insignificant contribution of genetic factors.^9^ Discrepancies in results could be due to reduced power, narrower age range and the use of computerised interview-based Composite International Diagnostic Inventory-Short Form instead of hospital registry data.

### Implication

This is the first study examining genetic overlap in the T2DM-depression comorbidity in two large, population-based twin registries. Our finding of qualitative genetic sex differences implies that although the comorbidity is mainly due to correlated genetic risk factors, these factors are not necessarily the same in males and females. Possible biological support for this observed latent effect is given by findings regarding the activation of the hypothalamic–pituitary adrenal axis, which has been postulated to have a major role in the T2DM and depression comorbidity.^25^ A preliminary study reported sex difference between hypothalamic–pituitary adrenal axis activity and adrenergic polymorphisms in 189 patients with depression.^26^ Increased hypothalamic–pituitary adrenal axis activity was associated with the ADRA2A genotype in males and ADRB2 genotype in females.

Other support for the observed qualitative sex differences in comorbidity is given by findings regarding visceral obesity, which has been suggested as a biological explanatory link between T2DM and depression comorbidity.^25, 27^ Anthropometric traits such as waist-to-hip ratio are well-established proxy measures of abdominal fat distribution.^28, 29^ Sex-specific dimorphic genetic effects have been reported for waist-to-hip ratios.^30^ Of particular interest, a female-only association was reported for the peroxisome proliferator-activated receptors region.^31^ Proliferator-activated receptors-γ has a well-established role in T2DM treatment and has been implicated in its pathology.^32^ It has also been postulated as a potential target for the treatment of depression, as it is closely related to parainflammation and endoplasmic reticulum stress, processes that can potentially interfere with normal stress response in a depressive illness.^33, 34^ Interestingly, the glucocorticoid receptor gene have been linked to both T2DM^35^ and depression,^36^ and its polymorphism has been associated with β-cell function in females, but not in males.^37^

The findings from this study have potential clinical relevance. Our study demonstrates that the T2DM-depression comorbidity may be genetic in origin, with different genetic factors being at play in males and females. Future research would involve developing sex-specific biopsychosocial explanatory models and conducting sex-specific analysis in molecular genetic studies. Understanding the underlying mechanism of the T2DM and depression comorbidity will provide useful insight into identifying potential biological mechanisms that may be accessible to interventions.

### Limitations

Our findings should be interpreted in the context of potentially significant limitations. No discrimination of types of diabetes was made in ICD-8 (1965) and the loss of participants at the time of retrieval of hospital registry data (2010 for the Swedish Twin Registry and 2011 for the Danish Twin Registry) may lead to misclassification of diabetes type. This is probably modest, given that T2DM comprises 90% of adults with diabetes.^38^ Estimations obtained from a single database should, however, be interpreted with caution.^39^ In addition, the mean age of onset for T2DM has been reported to be 46-year old^40^ and we have included all individuals aged over 18 in both samples. It is therefore possible that individuals without a diagnosis of T2DM still have the propensity to develop the illness.

Another important limitation of our study is the use of national health registries. The Swedish and Danish National Hospital Discharge Registries do not include visits to general practitioners and have been shown to capture more severe cases.^41^ Our constructs of T2DM and depression might therefore capture a more severe spectrum of the disorders that warrant outpatient care and/or hospital admission. In addition, there is a small possibility of misclassifications of cases as controls, such as when a participant had a past history of depression before the inception of the registry and did not subsequently have further episodes of depression requiring outpatient or inpatient care.

Studies have also shown that males were less likely to seek help.^42^ The prevalence rates for diabetes and depression were similar across the sexes (Table 1). Anthropometric traits such as body mass index have not been adjusted for in this study and we need to note the limitation that the observed genetic overlap can in part be due to the respective correlation of these traits with T2DM^43^ and depression.^44^ This and previous studies on T2DM and depression have focused on European-descent samples and replication in Asian and African descent populations are needed.

## Conclusion

This study uses two large, independent population-based twin samples to explore the genetic overlap between T2DM and depression. Our results suggest a genetic aetiology to the T2DM-depression comorbidity in males and females. It further suggests that different genetic risk factors may be involved in this comorbidity in males and females. T2DM and depression are chronic conditions with major public health impact. Understanding the pathophysiology of the T2DM and depression comorbidity, the basis for possible including sex differences, is critical for developing appropriate and effective interventions for this complex patient group.

## Figures and Tables

**Table 1:** Descriptive summary of MZ and DZ twins in Swedish and Danish twin samples, stratified by sex

*Sample*		*Swedish*		*Danish*	
		*Males*	*Females*	*Males*	*Females*
Number of paired twins Number of single twins	MZ	6524 2335	9270 2131	11 006 59	11 110 59
	DZ	7440 3757	9770 3421	19 538 130	17 640 113
	Opposite-sex	17 078 6880		34 900 848	
	Total	50 082 18 524		94 194 1209	
Age, mean (s.d.)	MZ	54.9 (17.9)	55.0 (18.7)	58.3 (16.8)	59.5 (18.3)
	DZ	60.3 (16.8)	61.3 (18.0)	60.5 (16.1)	63.0 (17.7)
	Opposite-sex	59.2 (16.5)		58.6 (15.7)	
	Total	58.5 (17.6)		60.0 (16.8)	
Prevalence of T2DM (%)^a^	MZ	5.5	4.2	4.1	3.3
	DZ	6.9	5.2	4.7	4.2
	Opposite-sex	5.8		3.1	
Prevalence of depression (%)^a^	MZ	3.7	5.8	2.8	4.7
	DZ	3.7	5.7	3.2	5.7
	Opposite-sex	4.9		3.8	
Proband wise concordance rate for T2DM (Number of concordant pair, discordant pair)	MZ	0.42 (69, 190)	0.43 (75, 195)	0.37 (83, 287)	0.33 (60, 247)
	DZ	0.20 (49, 398)	0.23 (53, 350)	0.14 (64, 764)	0.18 (66, 605)
	Opposite-sex	0.18 (91, 813)		0.13 (66, 886)	
Proband wise concordance rate for depression (Number of concordant pair, discordant pair)	MZ	0.21 (22,174)	0.22 (57, 399)	0.19 (29, 243)	0.23 (59, 400)
	DZ	0.06 (8, 249)	0.05 (13, 489)	0.05 (16, 591)	0.16 (77, 837)
	Opposite-sex	0.09 (37, 706)		0.09 (61, 1170)	

Abbreviations: DZ, dizygotic twins; MZ, monozygotic twins; T2DM, type 2 diabetes.

a In the genetic modelling, the thresholds and other estimates apply to the sample at mean age. For example, with the effect of age being estimated at −0.03 for males in the Swedish Twin Registry, T2DM prevalence rates will be 1.3% at age 40 and 9.0% at age 70.
