# Supplementary material for: Genetic overlap between type 2 diabetes and depression in Swedish and Danish twin registries
Source: Mol Psychiatry. 2016 Mar 29;21(7):903–9. doi: 10.1038/mp.2016.28 (PMC5414070; doi:10.1038/mp.2016.28)
Supplement: Supplementary Table 1 [file mp201628x1.docx]

**Supplementary Table 1.** International Classification of Disease (ICD) 8^th^, 9^th^ or 10^th^ Editions codes in full.

| **Codes** | **Diagnosis** |
| --- | --- |
| **ICD-8** |  |
| 250 | Diabetes mellitus |
| 296.0 | Affective psychoses - Involutional melancholia |
| 296.2 | Affective psychoses - Manic depressive psychosis, depressed type |
| 296.9 | Affective psychoses - Unspecified |
| 298.0 | Reactive depressive psychosis |
| 300.4 **Depressive neurosis** | Depressive neurosis |
| **ICD-9** |  |
| 250.00 | Diabetes mellitus without complication type II or unspecified type not stated as uncontrolled |
| 250.02 | Diabetes mellitus without complication type II or unspecified type uncontrolled |
| 250.10 | Diabetes with ketoacidosis, type II or unspecified type, not stated as uncontrolled |
| 250.12 | Diabetes with ketoacidosis, type II or unspecified type, uncontrolled |
| 250.20 | Diabetes mellitus with hyperosmolarity type II or unspecified type not stated as uncontrolled |
| 250.22 | Diabetes mellitus with hyperosmolarity type II or unspecified type uncontrolled |
| 250.30 | Diabetes mellitus with other coma type II or unspecified type not stated as uncontrolled |
| 250.32 | Diabetes mellitus with other coma type II or unspecified type uncontrolled |
| 250.40 | Diabetes mellitus with renal manifestations type II or unspecified type not stated as uncontrolled |
| 250.42 | Diabetes mellitus with renal manifestations type II or unspecified type uncontrolled |
| 250.50 | Diabetes mellitus with ophthalmic manifestations type II or unspecified type not stated as uncontrolled |
| 250.52 | Diabetes mellitus with ophthalmic manifestations type II or unspecified type uncontrolled |
| 250.60 | Diabetes mellitus with neurological manifestations type II or unspecified type not stated as uncontrolled |
| 250.62 | Diabetes mellitus with neurological manifestations type II or unspecified type uncontrolled |
| 250.70 | Diabetes mellitus with peripheral circulatory disorders type II or unspecified type not stated as uncontrolled |
| 250.72 | Diabetes mellitus with peripheral circulatory disorders type II or unspecified type uncontrolled |
| 250.80 | Diabetes mellitus with other specified manifestations type II or unspecified type not stated as uncontrolled |
| 250.82 | Diabetes mellitus with other specified manifestations type II or unspecified type uncontrolled |
| 250.90 | Diabetes mellitus with unspecified complication type II or unspecified type not stated as uncontrolled |
| 250.92 | Diabetes mellitus with unspecified complication type II or unspecified type uncontrolled |
| 296.2 | Major depressive disorder, single episode |
| 296.3 | Major depressive disorder, recurrent episode |
| 300.4 | Dysthymic disorder |
| 301.10 | Affective personality disorder, unspecified |
| 301.12 | Chronic depressive personality disorder |
| 301.13 | Cyclothymic disorder |
| 309.1 | Prolonged depressive reaction |
| 311 | Depressive disorder, not elsewhere classified |
| **ICD-10** |  |
| E11 | Type 2 diabetes mellitus |
| F32 | Major depressive disorder, single episode |
| F33 | Recurrent depressive disorder |
| F34.1 | Dysthymia |
| F38.1 | Other recurrent mood [affective] disorders |
