# Supplementary material for: Genetic overlap between type 2 diabetes and depression in Swedish and Danish twin registries
Source: Mol Psychiatry. 2016 Mar 29;21(7):903–9. doi: 10.1038/mp.2016.28 (PMC5414070; doi:10.1038/mp.2016.28)
Supplement: Supplementary Table 2 [file mp201628x2.docx]

**Supplementary Table 2. Tetrachoric correlation for i) type 2 diabetes, ii) depression and iii) type 2 diabetes – depression by zygosity and sex.**

| **Sample** |  | **Swedish** |  | **Danish** |  |
| --- | --- | --- | --- | --- | --- |
|  |  | **Males** | **Females** | **Males** | **Females** |
| **Within Trait** | **Cross Twin** |  |  |  |  |
| **T2DM** | MZ | 0.68  (0.60-0.75) | 0.72  (0.64-0.78) | 0.70  (0.63-0.76) | 0.65  (0.57-0.72) |
|  | DZ | 0.26  (0.16-0.36) | 0.39  (0.29-0.48) | 0.27  (0.19-0.34) | 0.36  (0.28-0.43) |
|  | Opposite-sex | 0.26  (0.19-0.33) |  | 0.28  (0.21-0.35) |  |
| **Depression** | MZ | 0.49  (0.36-0.60) | 0.45  (0.36-0.53) | 0.51  (0.41-0.61) | 0.50  (0.42-0.58) |
|  | DZ | 0.12  (-0.05-0.27) | -0.01  (-0.14-0.11) | 0.10  (-0.01-0.20) | 0.30  (0.23-0.37) |
|  | Opposite-sex | 0.20  (0.11-0.29) |  | 0.20  (0.13-0.26) |  |
| **Cross Trait** | **Cross Twin** |  |  |  |  |
| **T2DM-depression** | MZ | 0.02  (-0.10-0.13) | 0.12  (0.03-0.21) | 0.14  (0.05-0.23) | 0.10  (0.00-0.19) |
|  | DZ | 0.02  (-0.09-0.12) | 0.08  (-0.01-0.16) | 0.06  (-0.02-0.13) | 0.08  (0.02-0.14) |
|  | Opposite-sex | 0.02  (-0.05-0.08) |  | 0.07  (0.01-0.13) |  |

T2DM: type 2 diabetes; MZ: monozygotic twins; DZ: dizygotic twins

The MZ:DZ tetrachoric correlation ratios suggest ADE models for males and females for depression. However, since we are interested in the overall genetic overlap of depression and T2DM, we will only consider genetic effects in the broad sense.
